# Do kea parrots infer the weight of objects from their movement in a breeze?

**DOI:** 10.1098/rsbl.2024.0405

**Published:** 2024-11-06

**Authors:** Elizabeth Temeroli, Sarah A. Jelbert, Megan L. Lambert

**Affiliations:** ^1^ University of Exeter, Stocker Road, Exeter EX4 4PY, UK; ^2^ School of Psychological Science, University of Bristol, Bristol, UK; ^3^ Messerli Research Institute, University of Veterinary Medicine Vienna, Veterinaerplatz 1, Vienna 1210, Austria

**Keywords:** *Nestor notabilis*, kea parrots, New Caledonian crows, weight inference, causal inference

## Abstract

Weight, though it cannot be seen directly, pervades nearly every aspect of an animal’s life. However, the extent to which non-human animals reason about the property of weight remains poorly understood. Recent evidence highlights birds as a promising group for testing this ability: for example, New Caledonian crows can infer the weight of objects after observing their movements in a breeze. Here, we tested for similar weight inference abilities in kea (*Nestor notabilis*), a parrot species known for its sophisticated problem-solving skills. Subjects were trained to exchange objects of a target weight (light or heavy) for a food reward. They were then allowed to observe pairs of novel objects (one light and one heavy) hung in front of an electric fan in both an experimental condition (fan on, light object moving) and a control condition (fan off, both objects motionless). The birds were subsequently presented with test trials in which they could use the information from the demonstration to select an object of their target weight. We found that, unlike New Caledonian crows, kea did not perform significantly better on trials in which they observed the objects’ movements and discussed our findings within the context of the kea’s highly explorative nature.

## 1. Introduction

Weight is a fundamental property of objects, impacting our daily lives and influencing our decision-making processes. For example, we know to use a stone, rather than a leaf, to keep the picnic blanket from flying away on a windy day. Non-human animals, too, often face challenges involving weight: based on weight, capuchin monkeys select what nuts to crack open and what stones to do it with, while passerine birds use weight information to decide which seeds to eat [1–3]. While in these instances, animals rely on the handling of objects to establish their weight, from infancy, humans can make inferences about weight based on environmental cues [4]. Indeed, when observing the wind blow away the leaf and not the stone, we know that the stone is heavier than the leaf without needing to test this knowledge by picking up either.

While some scholars have argued, following the results of primate studies, that such an understanding of weight is likely limited to humans [5], other research has provided evidence of the contrary. Chimpanzees (*Pan troglodytes*) locate hidden food based on its positioning on a balance beam, likely inferring that the additional weight of the food caused the beam to tilt ([6], but see [5]). More recently, New Caledonian crows (*Corvus moneduloides*) were shown to infer the weights of unfamiliar objects after observing their movements in a breeze ([7], but see [8]). Specifically, birds that had been trained to choose between pairs of objects based on their weight correctly selected *novel* objects of their target weight more often than chance if they first observed the novel objects hanging in front of an electric fan (which caused light but not heavy, objects to sway). This contrasted with chance performance in a control condition where the fan was off [7]. As corvids and primates last shared an ancestor 324 million years ago [9], these findings suggest a case of convergent evolution of complex physical reasoning skills. However, the degree to which other species make inferences about weight remains mostly untested. It may be an ability shared among many avian and mammalian species or an example of sophisticated reasoning, unique to primates and the tool-making New Caledonian crows.

We aimed to elucidate causal weight understanding in a large-brained parrot species: the kea (*Nestor notabilis*). Kea are omnivorous parrots native to the alpine areas of New Zealand [10]. While instances of tool use have been observed in kea following facilitation in captive and wild settings, unlike New Caledonian crows, they are not known to habitually rely on tools [11–15]. Nonetheless, their cognitive abilities are in many ways comparable to those of primates and corvids [12,15,16], and their exploratory nature makes them ideal subjects for physical cognition research [17]. In a previous study examining the function of object exploration in kea and New Caledonian crows, both species appeared to learn about the weights of objects during exploration and used this information to select the right tool to release food from a container [15].

Here, we adapted the methodology utilized by Jelbert *et al*. [7] to explore whether kea can use causal environmental cues to reason about the weight of objects. We hypothesized that, if such inferences are commonplace among birds, then like crows, kea would use the behaviour of objects in a breeze to infer the objects’ weight and subsequently utilize this information in an object-choice task.

## 2. Methods

This study was pre-registered on the Open Science Framework (https://osf.io/tu8nk).

### (a) Subjects

Subjects were eight captive adult kea parrots (four males and four females; see electronic supplementary material, table S1). The parrots were housed socially in a large outdoor aviary (520 m^2^) at the Haidlhof Research Station in Bad Vöslau, Lower Austria.

### (b) Materials

**Figure 1.**
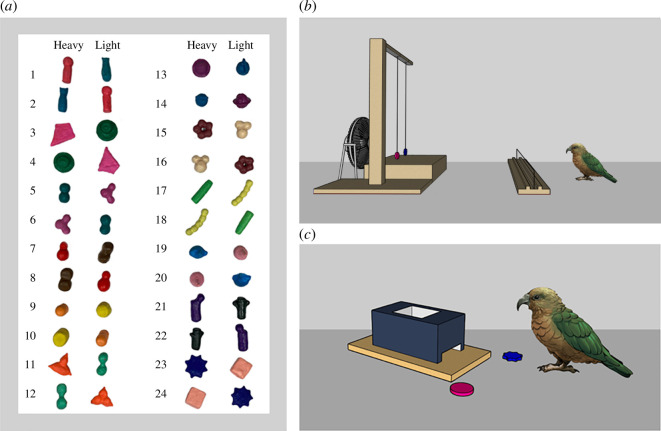
Experimental set-up and materials. (*a*) The object pairs used in the study and (*b*) set-up during the observational phase. The barrier between the bird and the fan represents the aviary’s outer mesh wall. (*c*) Set-up during the choice phase.

Throughout the study, the birds were presented with pairs of visually distinct novel objects (figure 1*a*). Object pairs consisted of one light (16 g) and one heavy (116 g) object. All objects were made from polymer clay, with heavy objects containing hidden non-toxic fishing weights (see electronic supplementary material for more details). Kea had to place objects inside a wooden box to receive food rewards (one-eighth of a peanut). The box was remote-controlled so that when birds dropped an object inside, the experimenter could press a button to release the reward.

### (c) Procedure

Testing took place between October and December 2022. The parrots followed their usual diets and had ad libitum access to water. Subjects were tested once per day, between feeding times. All sessions were conducted in visually isolated compartments attached to the main aviary. Test sessions were video recorded.

#### (i) Training

Subjects were first trained to drop objects of a certain weight (either 16 g, *light-rewarded*: four birds, or 116 g, *heavy-rewarded*: four birds) into a box to obtain food rewards. In an earlier experiment on learning speeds, kea were presented with pairs of visually identical clay objects (two grey cuboids, weighing 16 g and 116 g) to learn this rule. Here, to generalize the rule to new objects, kea were presented with pairs of visually distinct objects (light: 16 g and heavy: 116 g). Birds could contact both objects, and trials ended when they dropped one into the box. Kea passed the training when they chose the object of their target weight on 9/10 trials within a session. All birds succeeded within one to three sessions (see electronic supplementary material for full training procedure).

#### (ii) Test

The experiment properly assessed whether kea used information from the movement of objects in the breeze to guide their selection of novel objects. Birds received six test sessions in an experimental condition and six in a control condition in an alternating pattern, counterbalanced across birds. Each session used a different pair of novel objects and comprised two phases: an observational phase and a choice phase (electronic supplementary material, video S1). In the observational phase, an object pair was tied to strings and positioned in front of an electric fan located outside the aviary so that the birds could see but not interact with the objects (figure 1*b*). Observational phases lasted 5 min, during which time kea could roam freely in the test aviary with full visual access to objects and fan. In the experimental condition, the fan was switched on, allowing the bird to witness the objects’ movements and experience the breeze coming from the fan. In the control condition, the fan was switched off, and the objects were motionless.

When the observational phase ended, the objects were detached from the strings and placed inside the enclosure next to the food-dispensing box (figure 1*c*). Birds then immediately began a choice phase where they were allowed to freely interact with both objects. All object contacts were recorded, and birds were rewarded if they dropped the object that matched their target weight into the box. Once birds had dropped an object into the box, the trial ended. The experimenter retrieved both objects and replaced them next to the box, randomizing their position before allowing the bird to choose between the objects again, for a total of five trials per object pair.

Our primary interest lay in whether the birds *contacted* the correct object first—on their first trial with each novel pair—more often than chance in the experimental condition but not in the control condition. This pattern of results would suggest that kea could use information gained from observing the objects’ movements in a breeze to inform their choices. We also recorded which object they *dropped* into the box on their first trial, which object they contacted first on each of the five trials with each object pairing and which object they dropped into the box across the five trials with each pair.

### (d) Analysis

Data were analysed in R (v. 3.6.0; [18]). We ran one-sample Wilcoxon signed-rank tests with continuity corrections to determine whether the proportion of first trials in which birds (i) touched the correct object first and (ii) dropped the correct object first varied significantly from chance (*μ* = 0.5) in each condition.

To estimate the effect of condition on the binary outcomes of first contact (correct or incorrect, GLMM1) and final choice (correct or incorrect, GLMM2), we fitted generalized linear mixed models (GLMM; [19]) with a binomial error structure and logit link function using the function lmer of the package lme4 [20]. We included condition as a fixed effect, along with the control variables target weight, session and trial, with the latter two *z*-transformed. The subject was included as a random intercept effect (see electronic supplementary material for further details).

## 3. Results

**Figure 2.**
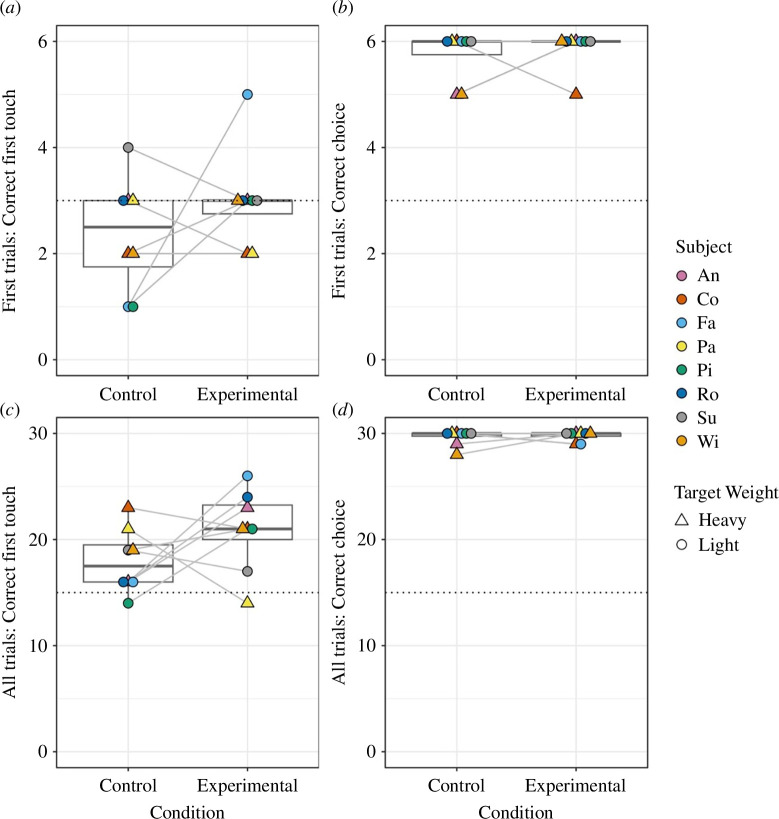
Performance between conditions. In each condition (control = fan off; experimental = fan on), six novel object pairs were used, and each pair was presented five times in succession (six object pairs × five trials per pair, per condition). In all trials, birds could touch either object and then drop one object into the apparatus to obtain a reward. Panels (*a*) and (*b*) depict the number of times subjects (*a*) touched the correct object first and (*b*) dropped the correct object into the box on their *very first trial* with each novel object pair. Panels (*c*) and (*d*) depict the number of times subjects (*c*) first touched and (*d*) dropped the correct object into the box *across all trials* (five trials per object pair). Grey lines indicate paired samples, the thick black line denotes the median, the box denotes the interquartile range (IQR) between 25th and 75th percentile and whiskers show a range of data within IQR × 1.5. Dotted line represents the chance level.

Contrary to our predictions, birds *touched* the correct object at chance levels on their first trial with each novel object set in the experimental condition (fan on: 24/48, 50% correct, one-sample Wilcoxon test: *V* = 3, *p* = 1) as well as the control condition (fan off: 19/48, 40% correct, *V* = 1, *p* = 0.1025; figure 2). The birds selected the correct object to *drop* into the box from their first trial with near-perfect accuracy in both conditions (experimental: 47/48, 98% correct, *V* = 36, *p* = 0.008; control: 46/48, 96% correct, *V* = 36, *p* = 0.01).

The sample size for both GLMMs consisted of a total of 480 trials conducted in 96 sessions with eight individuals. We found no significant effect of condition on whether the birds first contacted the correct object (full-null model comparison: *χ^2^* = 1.775, d.f. = 1, *p* = 0.187; electronic supplementary material, table S2) or whether they ultimately dropped the correct object into the box (full-null model comparison: *χ^2^* = 0.228, d.f. = 1, *p* = 0.637; electronic supplementary material, table S2). To address potential concerns about limited power arising from our relatively small sample size, we report our confidence intervals in electronic supplementary material, table S2 as a suggested alternative to *post hoc* power analysis [21–23]. Specifically, confidence intervals reflect a range of effect sizes based on the observed data, likely containing the true effect size, the breadth of which allows for an assessment of the uncertainty surrounding the effect size [21–23].

## 4. Discussion

Overall, our results suggest that, unlike New Caledonian crows [7], kea do not appear to use the movement of objects in a breeze generated by an electric fan as a cue to infer the objects’ weight. Contrary to our predictions, kea’s choice of which object to approach first did not significantly differ from chance in either the experimental (fan on, light object moving) or the control (fan off, both objects motionless) conditions. All subjects were accurate in choosing which object to ultimately put into the box, dropping an incorrect object in just five out of 480 trials. This was true independent of condition and regardless of which object they contacted first, suggesting that (i) they were relying on the objects’ weights while making their choice, but (ii) direct manipulation appears to have been required for them to gain weight-related information in this study.

Our findings, therefore, do not support the hypothesis that inferring weight via observation of causal contextual cues is a broadly shared ability among birds, and we tentatively suggest that making such inferences could reflect a specialism of New Caledonian crows, already known for their impressive tool-manufacture [24] and problem-solving across a range of domains [12,16,25]. Though, below, we briefly note alternative explanations to consider.

Here, we closely followed Jelbert *et al*.’s [7] methodology, but with modifications owing to species and testing facility. First, to account for New Caledonian crows’ neophobia, the crows were exposed to the fan and objects for three short observational phases in the morning before receiving a test session (comprising a final observational and choice phase) in the afternoon. Here, kea received the test session comprising a 5-min observational and choice phase only. As a highly neophilic species, kea tend to be more interested in objects upon first exposure; thus, a single observational phase was chosen to provide the kea with ample time to approach and observe the objects, while minimizing the risk of demotivation due to repeated inactivity in the test compartment. It is possible that kea may have performed better if allowed further observation time, though our results do not suggest this to be likely. Second, the interval between the observational and choice phases was slightly longer for kea than crows (both less than 5 min) due to differences in enclosures. During said interval, birds had a full view of the testing compartment and could observe the experimenter remove the objects from the fan and place them in front of the box. Kea perform well in various other complex cognitive tasks [17] and can track the movement of hidden objects [26]; however, we cannot rule out that kea *can* make inferences about weight but failed to demonstrate this here due to the task’s memory demands.

Other researchers have argued that the crows’ successes can be best described as evidence that these birds can represent a relationship between ‘effort-to-lift’ (i.e. how strenuous it feels to pick up certain objects) and ‘spontaneous-displacement-of-objects’ (i.e. how much objects move in a breeze) [8]. This relationship would have been learnt through prior experience of interacting with and observing different objects. The complete past experiences of both the wild-caught crows and captive kea (housed in large and enriched outdoor aviaries) are unknown, though we can be certain that both were exposed to natural weather conditions and free-moving objects. We agree that prior experience is essential; thus, the differences we observed among crows and kea might reflect their prior exposure to, or their *attention to*, the movement of objects by the wind.

Indeed, a related explanation for our results, which we believe is compelling, could be found in the kea’s neophilia [10,27]. As exploratory foragers, kea may be more reliant on the haptic manipulation of objects to glean their qualities rather than visual inspection, which, conversely, may be more significant for neophobic crows. Previous research exploring flexible tool use among kea and New Caledonian crows found that crows engaged in visual inspections of provided objects, while kea explored objects via direct contact [12]. In the same study, kea were ultimately quicker than crows to master different tools despite the crows being natural tool users [12]. Other comparative studies also found kea consistently relied on physical exploration more than corvids and demonstrated similar rates of problem-solving successes [15,28]. As kea inhabit a seasonal island environment where predation risk and competition are low, physical exploration—and the quick and accurate problem-solving this enables—may be particularly advantageous [10,27,29], negating the benefits of acquiring information via inference. To clarify this, future research could explore whether kea use other visual cues to infer weight, such as size or material, as used by humans and primates [18,19,30–33].

In conclusion, kea, unlike New Caledonian crows, do not appear to infer weight from the movement of objects in a breeze. The kea’s neophilia may promote haptic exploration over the drawing of causal inferences via observation. Given the dearth of research in this area, further work is necessary to uncover the role of ecology in inferential reasoning abilities and their distribution among birds.

## Data Availability

The data are provided in the electronic supplementary material, which is available online [34].
